# Comparison of the Effect of the Combination of Sodium Valproate and Sodium Dichloroacetate on the Expression of *SLC12A2*, *SLC12A5*, *CDH1*, *CDH2*, *EZH2*, and *GFAP* in Primary Female Glioblastoma Cells with That of Temozolomide

**DOI:** 10.3390/pharmaceutics17091161

**Published:** 2025-09-04

**Authors:** Dovydas Gečys, Laimis Akramas, Aidanas Preikšaitis, Ingrida Balnytė, Arūnas Vaitkevičius, Julija Šimienė, Donatas Stakišaitis

**Affiliations:** 1 Laboratory of Molecular Cardiology, Institute of Cardiology, Lithuanian University of Health Sciences, 50161 Kaunas, Lithuania; dovydas.gecys@lsmu.lt; 2 Dca Farma, UAB, 47296 Kaunas, Lithuania; 3 Clinic of Neurology and Neurosurgery, Center of Neurosurgery, Faculty of Medicine, Vilnius University, 03101 Vilnius, Lithuania; aidanas.preiksaitis@santa.lt; 4 Department of Histology and Embryology, Medical Academy, Lithuanian University of Health Sciences, 44307 Kaunas, Lithuania; ingrida.balnyte@lsmu.lt; 5 Clinic of Neurology and Neurosurgery, Faculty of Medicine, Institute of Clinical Medicine, Vilnius University Hospital Santaros Klinikos, Vilnius University, 08661 Vilnius, Lithuania; arunas.vaitkevicius@santa.lt; 6 Laboratory of Molecular Oncology, National Cancer Institute, 08660 Vilnius, Lithuania; julija.simiene@nvi.lt

**Keywords:** KCC2, NKCC1, CDH1, CDH2, EZH2, GFAP, glioblastoma, primary cells, IDH-wild-type, temozolomide, sodium valproate, sodium dichloroacetate

## Abstract

The search for an effective treatment for adult high-grade glioblastoma (GBM) remains urgent. **Background/Objectives**: The study aimed to determine the expression of carcinogenesis-related genes, such as *SLC12A2*, *SLC12A5*, *CDH1*, *CDH2*, *EZH2*, and *GFAP*, in primary glioblastoma (WHO Grade IV; IDH-wild-type) cells from three adult women: GBM5-1, GBM5-2F, and GBM5-3F. **Methods**: The impact of the combination of sodium valproate and sodium dichloroacetate (2 mM NaVPA–3 mM NaDCA) on the expression of these genes was determined and compared with the effects of 50 µM temozolomide after 24 h of treatment. **Results**: 2 mM NaVPA–3 mM NaDCA, as well as temozolomide, had individual impacts on the *SLC12A2*, *SLC12A5*, *CDH1*, *CDH2*, *EZH2*, and *GFAP* expressions of tested GBM5-1, GBM5-2F, and GBM5-3F primary cells of female GBM patients. **Conclusions**: The combination of 2 mM NaVPA–3 mM NaDCA may have an advantage in antitumor activity and may be more effective than TMZ; however, the effect is individual.

## 1. Introduction

Adult diffuse glioblastoma (GBM) is the most aggressive brain tumor type with the worst prognosis; high-grade glioblastoma has a 5-year overall survival (OS) of 5% of patients [1]. GBMs (WHO Grade IV) are classified as primary brain tumors, as IDH-wild-type GBMs, which represent the most frequent malignant brain tumors, with an OS ranging from 12 to 18 months [2]. Women are slightly less likely to suffer from GBM and have a slightly better median survival than men [3]. GBM is a highly heterogeneous tumor in which different cell types coexist, including tumor cells, fibroblasts, endothelial cells, and immune cells [4,5,6]. GBM subtypes can coexist in different tumor parts and within their cells [7]. Differences in tumor tissue are found between different stages, genders, and age groups. High heterogeneity of tumor cells is associated with invasive and metastatic manifestations [8].

The approach to GBM treatment to date is to personalize each clinical case, considering the diagnosis time, onset or relapse, age, tumor cancer markers, and the patient’s general condition. The current standard treatment approach for newly diagnosed GBM involves tumor-maximal surgical resection with radiotherapy and temozolomide (TMZ) therapy [9]. Chemotherapy is a key part of the treatment regimen for adult patients [10]. TMZ was registered in 2005 for the treatment of newly diagnosed GBM [11,12]. Treatment of GBM after surgery with the addition of TMZ to radiotherapy has increased OS by 2 months [13], with a median OS of 15 months and progression-free survival of 6 months. In more than 50% of patients with GBM, TMZ treatment is ineffective [14,15]. TMZ is only helpful in patients with GBM with a methylated-DNA-protein-cysteine methyltransferase (MGMT) promoter, which is slightly more common in women than in men, supporting their better survival outcomes, but the toxic effects of chemotherapeutic agents are more common in women [16]. Thus, there is currently no effective treatment for GBM, and the search for one, especially for individualized treatment with higher efficacy and lower toxicity, is urgent.

The primary effects of drugs on GBM are related to the inhibition of tumor cell proliferation and the activation of apoptosis [17]. Activated glycolytic processes characterize GBM cells [18]. Tumor cells exhibit increased expression of pyruvate dehydrogenase kinases (PDKs), leading to enhanced glycolytic activity in GBM cells [19,20]. Consequently, inhibition of PDKs is a target for GBM treatment [21].

As an anticancer preparation, sodium dichloroacetate (NaDCA) selectively acts on cancer cells by inhibiting PDK [22], thereby activating pyruvate dehydrogenases (PDHs), which reduces lactate levels in both the blood and the tumor microenvironment, inhibiting tumor growth and spread [23]. Preclinical studies have shown that NaDCA salts are associated with a reduction in tumor cell proliferation rate and metastatic spread, and activation of apoptosis [22]; NaDCA inhibits U87 MG and PBT24 glioblastoma xenografts growth, frequency of tumor invasion, and the number of blood vessels in the chicken chorioallantoic membrane model; NaDCA impacts on tumor proliferating cell nuclear antigen (PCNA) and enhancer of zest homolog 2 (EZH2) expression in the tumor tissue cells [24]. Studies of immunodeficient mice with U87 tumors in the brain have shown that the combination of NaDCA and radiotherapy increases the survival of the mice [25].

Sodium valproate (NaVPA) is a histone deacetylase (HDAC) inhibitor, specifically targeting classes I and IIa, and an epigenetic modulator that affects gene expression by inhibiting tumor cell proliferation and inducing apoptosis [26,27]. HDACs may regulate GBM progression. HDAC2 expression is upregulated in GBM cells. Knockdown of HDAC2 inhibits GBM cell proliferation and invasion, suggesting that HDAC2 may be a potential target for treating GBM and enhancing the efficacy of TMZ therapy [28]. HDAC2 knockdown inhibits glioblastoma tumorigenesis through regulating glucose metabolism and proliferation [29]. NaVPA alone or combined with other therapies inhibits glioma growth in vivo and in vitro [30]. Non-toxic doses of NaVPA increase the sensitivity of U87 and T98G cells to gefitinib by inhibiting cell growth through the activation of autophagy [31]. NaVPA binds E2F transcription factor 1 to the glycosylated GPI and PGK1 promoter to inhibit glycolysis [32]. NaVPA may enhance the transport of the dichloroacetate anion into the cell via mitochondrial mechanisms [33,34]. The synergistic effects of chemotherapy and HDAC inhibitors are promising for treating GBM and preventing chemotherapy resistance [35].

The inflammatory microenvironment of the GBM tumor, characterized by the release of pro-inflammatory cytokines and chemokines, as well as the activation of inflammatory pathways, promotes tumor malignancy, which is associated with increased resistance of GBM to therapy [36,37]. Changes in the microenvironment of cancer tissues are characterized by increased lactic acid content [38]. NaDCA is an investigational antitumor drug with anti-inflammatory properties [39]. NaVPA’s immunomodulatory and anti-inflammatory effects are also well-established [40,41]. Treatment with NaVPA–NaDCA increased *Slc5a8* expression in mouse thymocytes, indicating an effect of NaVPA on the dichloroacetate anion transporter, and significantly affected the expression of genes related to inflammation and immune response [42]. Experimental research data, both in vivo and in vitro, indicate that NaVPA–NaDCA exhibits a more effective anticancer effect on GBM compared to NaDCA, and its efficacy surpasses that of TMZ. However, the effects of the investigational drugs on GBM differ depending on the cell line [43]. NaVPA–NaDCA inhibits inflammatory and immune response pathways [44], which are also characteristic of GBM.

Understanding the mechanisms of action of drugs and the evolution of resistance to treatment is crucial for the efficacy of GBM treatment, as it enables the assessment of the impact of drugs on changes in cancer prognostic markers [45,46]. Advances in the personalized treatment of GBM are closely tied to standard treatment, and new treatment strategies are being explored, including chemotherapy with drugs targeting the molecular and metabolic properties of the tumor [47,48]. Tumor cell proliferation and epithelial–mesenchymal transition (EMT) are central regulators of GBM invasion and are regulated by several factors that determine and promote cancer progression, related to the glycolytic phenotype of cells, which is closely linked to GBM malignancy [49]. The Na-K-2Cl cotransporter (NKCC1, encoded by *SLC12A2*) increases intracellular Cl^−^ concentration and enhances GBM cell proliferation [50,51,52], and it is involved in the EMT process. The K-Cl cotransporter (KCC2; encoded by *SLC12A5*) regulates the efflux of K^+^ and Cl^−^ ions from the cell. Apoptosis requires a loss of cell volume, which occurs through the reduction in intracellular [K^+^]i and [Cl^−^]i, before any other detectable features of apoptosis are observed. Loss of [K^+^]i and [Cl^−^]i in the glioma cell in parallel with expressed apoptosis was confirmed [53,54,55]. A EMT in part is a result of the downregulation of E-cadherin (*CDH1*) and the parallel upregulation of N-cadherin (*CDH2*) *genes* [56]. Downregulation of *CDH1* might be related to the induction of EMT [57]. It was reported that by regulating *SLC12A5* expression, EZH2 activates the WNK1-OSR1-NKCC1 (lysine-deficient protein kinase-1—oxidative stress responsive 1—NKCC1) pathway to promote glioma migration and tumor invasion, while also promoting *SLC12A5* DNA methylation [58]. EZH2’s role in regulating *GFAP* (glial fibrillary acidic protein) expression may offer insights into the molecular mechanisms of GBM [59,60]. Understanding this interplay could elucidate the pathophysiology of *GFAP* expression and help develop strategies for targeting GBM progression [60]; therefore, the exact mechanism in GBM needs further exploration.

The study aimed to determine the differences in the primary cell response of IDH-wildtype GBMs to treatment with the sodium valproate and sodium dichloroacetate combination (NaVPA–NaDCA) by assessing the expression of cancer marker genes *SLC12A2*, *SLC12A5*, *CDH1*, *CDH2*, *EZH2*, and *GFAP*, and to compare these effects with those of TMZ.

NaVPA–NaDCA and TMZ had individual effects on the expression of *SLC12A2*, *SLC12A5*, *CDH1*, *CDH2*, *EZH2*, and *GFAP* in GBM5-1, GBM5-2F, and GBM5-3F primary cells. The study suggests that the NaVPA–NaDCA combination may have an anticancer activity advantage over TMZ, but the effect depends on the patient’s cells. It is essential to assess the sensitivity of GBM cells to the drug before initiating chemotherapy when determining a treatment strategy.

## 2. Materials and Methods

### 2.1. GBM Patient Clinical Data

The inclusion criteria for study patients were as follows: adult patients with a first diagnosis of GBM, who had not received anticancer drugs and were not using valproic acid. GBM tissue samples were obtained from three patients who underwent surgery, and their details are provided below. GBM5-1F, 45-year-old female; glioblastoma diffuse (WHO Grade IV), IDH-wild-type; tumor of the left-sided frontoparietal region with spread to the basal ganglia and upper part of the brain stem; strong positive cytoplasmic reaction in GFAP tumor cells (surgery 28 November 2024); GBM5-2F, 77-year-old female; GBM diffuse (WHO Grade IV), IDH-wild-type; left frontal region tumor; strong positive cytoplasmic reaction in GFAP tumor cells (surgery 6 January 2025); GBM5-3F, 83-year-old female; GBM diffuse (WHO Grade IV), IDH-wild-type; tumor of the left parietoccipital area; strong positive cytoplasmic reaction in GFAP tumor cells (surgery 24 March 2025). The Vilnius Regional Biomedical Research Ethics Committee granted authorization to conduct biomedical research on 11 November 2024, under authorization No. 2024/11-1625-1077. Patients were operated on at Vilnius University Santaros Hospital Neurosurgery Clinic (Santariškių St. 2, Vilnius); patients were not treated with anticancer drugs prior to surgery.

### 2.2. Preparation of Cells for Analysis from Tumor Tissue

The explanted tumor tissue was placed in Ca^2+^-depleted Dulbecco’s Modified Eagle Medium (Thermofisher Scientific, Gibco, Roterdam, The Netherlands) supplemented with 1% penicillin-streptomycin (Thermofisher Scientific, Gibco, Roterdam, The Netherlands); the time taken to harvest the tumor for analysis after tumor removal was no longer than 10 min, and the tissue was kept at 37 °C in the medium. In a Ca^2+^-free medium, the tissue was dissected into small pieces using needle tweezers and then gently pipetted to obtain a cell suspension. Suspension was centrifuged at 110× *g* for 5 min, and the supernatant was discarded. Cells were seeded into 6-well multiwell tissue culture plates (TPP, Trasadingen, Switzerland) and grown in depleted Dulbecco’s Modified Eagle Medium (Thermofisher Scientific, Gibco, Roterdam, The Netherlands) supplemented with 10% fetal bovine serum (Thermofisher Scientific, Gibco, Roterdam, The Netherlands) and 1% penicillin-streptomycin (Thermofisher Scientific, Gibco, Roterdam, The Netherlands) under standard conditions at 37 °C and 5% CO_2_. Experimental groups comprised control, NaVPA–NaDCA, and TMZ groups, in which cells were treated with a phosphate-buffered saline (Thermofisher Scientific, Gibco, The Netherlands), 2 mM NaVPA, and 3 mM NaDCA (Sigma Adlrich, Steinheim, Germany) combination or 50 µM TMZ (Sigma Aldrich, Steinheim, Germany), respectively, for 24 h. After incubation, cells were scraped, transferred into a sterile 15 mL tube, and centrifuged at 110× *g* for 5 min. The supernatant was discarded, and fresh cell pellets were used to extract the RNA.

### 2.3. Total RNA Extraction and Real-Time qPCR

Total RNA was extracted from the cells using the TRIzol™ Plus RNA Purification Kit (Invitrogen™, Thermo Fisher Scientific, Roterdam, The Netherlands). The concentration of the extracted RNA was measured with a NanoDrop 2000 spectrophotometer (Thermo Scientific, Waltham, MA, USA). RNA was reverse transcribed into cDNA using the High-Capacity cDNA Reverse Transcription Kit (Applied Biosystems™, Thermo Fisher Scientific, Roterdam, The Netherlands), with the addition of RNaseOUT™ Recombinant Ribonuclease Inhibitor (Invitrogen™, Thermo Fisher Scientific, Roterdam, The Netherlands). Real-time PCR for *SLC12A2* (TaqMan Assay ID: Hs00169032_m1), *SLC12A5* (TaqMan Assay ID: Hs00221168_m1), *CDH1* (TaqMan Assay ID: Hs01023895_m1), *CDH2* (TaqMan Assay ID: Hs00983056_m1), *EZH2* (TaqMan Assay ID: Hs00544830_m1) and *GFAP* (TaqMan Assay ID: Hs00909233_m1) were carried out using TaqMan chemistry (Applied Biosystems™, Thermo Fisher Scientific, Roterdam, The Netherlands) following the manufacturer’s protocol on a 7900 Real-Time PCR System (Applied Biosystems™, Thermo Scientific, Carlsbad, CA, USA). All reactions were performed in triplicate. The glyceraldehyde-3-phosphate dehydrogenase gene (*GAPDH*) was used as the reference gene (TaqMan Assay ID: Hs02786624_g1).

### 2.4. Investigational Medicinal Preparations

Investigational medicinal preparations used for the study were sodium valproate (NaVPA; Sigma-Aldrich, Steinheim, Germany), sodium dichloroacetate (NaDCA; Sigma-Aldrich, Steinheim, Germany), and temozolomide (Sigma-Aldrich, St. Louis, MO, USA). The combination of NaVPA and NaDCA is patented: a patent filed that covers VPA–NaDCA as a new medicinal product for the treatment of cancer (Official bulletin of the State Patent Bureau of the Republic of Lithuania, No. 6874, filling date 17 April 2020); a European patent application has been submitted (European patent application No. 21168796.7, filing date 16 April 2021). Primary GBM cells were treated with 2 mM NaVPA and 3 mM NaDCA combination (NaVPA–NaDCA) for 24 h. The doses of the investigational drugs were selected based on studies conducted in vivo and in vitro with GBM cell lines [43]. The selected TMZ concentration for cell treatment corresponds to the average blood plasma concentration in patients treated with TMZ [61].

### 2.5. Statistical Analysis

Statistical analysis and graphs were generated using GraphPad Prism 9 software (GraphPad Software, Inc., San Diego, CA, USA). For statistical purposes, the threshold cycle (CT) values of single PCR replicates were normalized to the control *GAPDH* for gene expression analysis, and the ΔCT value was calculated, which was further used for statistical tests. The Shapiro–Wilk test was used to verify the normality assumption. Most of the samples had a Gaussian distribution; however, due to the small sample size within groups, non-parametric tests were used. The difference between the two independent groups was evaluated using the Mann–Whitney *U* test. Kruskall–Wallis test with Benjamini, Krieger, and Yekutieli multiple comparisons correction was used to determine differences between the medians of three independent groups (Tables S1–S6). The Livak (2^−ΔΔCT^) method was used to calculate the expression between the treated and control groups of the investigated genes. The alterations in gene expression are presented as percentages and were calculated from fold change values, assuming that a fold change of 2 represents a 100% increase and a fold change of 0.5 corresponds to a 50% decrease in gene expression. Statistical significance was defined as *p* < 0.05.

## 3. Results

The data from the gene expression assays are tabulated in the Supplementary Materials: Tables of Supplementary Data (Tables S1–S6), which presents the tested gene expressions in control cells and the gene expression after treatment with the test drugs compared to the control.

### 3.1. The Effect of NaVPA–NaDCA or TMZ on the SLC12A2 Expression in Female GBM Patients’ Tumor Primary Cells

**Figure 1:**
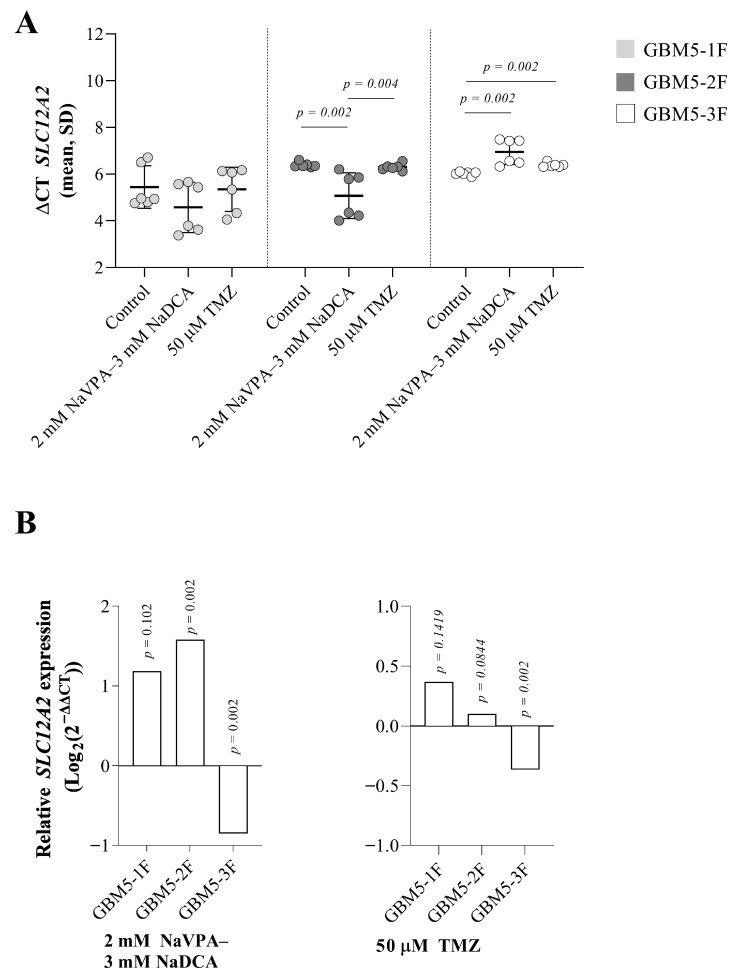
(**A**) The *SLC12A2* expression (ΔCT) of the GBM patient tumor control group, normalized to the *GAPDH*. (**B**) Relative *SLC12A2* expression (Log_2_(2^−ΔΔCT^)) of female GBM patient tumor groups tested. Exact *p*-values are given compared to the control.

Figure 1 presents the *SLC12A2* expression data of the tested primary GBM cells (control and treated with NaVPA–NaDCA or TMZ) from women who underwent surgery.

There was no difference in *SLC12A2* expression in GBM-1F control cells compared to GBM5-2F control and GBM5-3F control cells, but when comparing the latter two, GBM5-2F cells had significantly lower gene expression than GBM5-3F cells (*p* = 0.002). Figure 1B depicts significant relative *SLC12A2* expression differences between GBM5-2F- and GBM-3F-treated and their control groups.

Treatment with 2 mM NaVPA–3 mM NaDCA significantly increased the expression of *SLC12A2* in GBM5-1F and GBM5-2F cells (130% and 200%, respectively), but upregulation of *SLC12A2* was not significantly different between GBM5-1F and GBM5-2F tumor cells after treatment with NaVPA–NaDCA. Combination treatment significantly reduced gene expression by 45% in GBM5-3F cells compared to the control. TMZ reduced *SLC12A2* expression in GBM5-3F cells by 22% compared to the control, but did not affect *SLC12A2* expression in other patient cells treated with TMZ (Figure 1B).

### 3.2. The Effect of NaVPA–NaDCA or TMZ on the SLC12A5 Expression in Female GBM Patients’ Tumor Primary Cells

The expression of *SLC12A5* was significantly different in all control cells tested (*p* = 0.002). Specifically, GBM5-3F cells exhibited the highest gene expression (*p* = 0.002), while GBM5-1F cells had significantly lower expression than GBM5-3F cells (*p* = 0.009).

**Figure 2:**
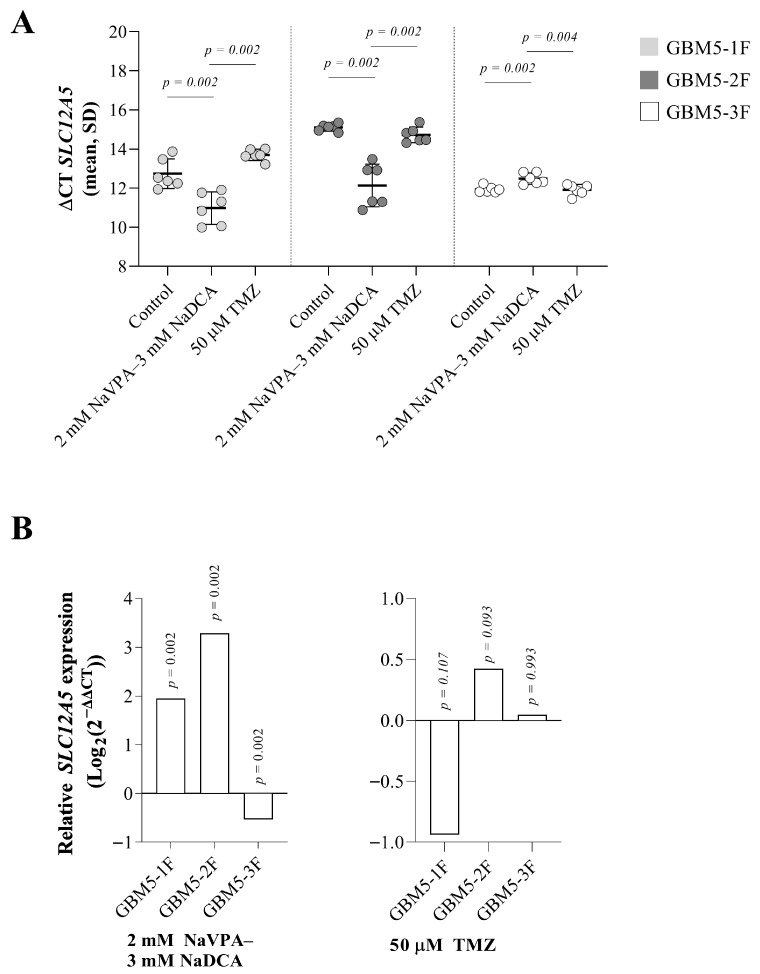
(**A**) The *SLC12A5* expression (ΔCT) of the GBM patient tumor control group was normalized to the *GAPDH*. (**B**) Relative *SLC12A5* expression (Log_2_(2^−ΔΔCT^)) of female GBM patient tumor groups tested. Exact *p*-values are given compared to the control.

Figure 2A demonstrates that *SLC12A5* expression increased in GBM5-1F and GBM-2F cells but decreased in GBM5-3F cells after NaVPA–NaDCA treatment. TMZ had no impact on gene expression. All cells treated with the combination showed higher *SLC12A5* expression than those treated with TMZ.

Compared to the control, NaVPA–NaDCA treatment significantly increased relative *SLC12A5* expression in GBM5-1F and GBM5-2F (by 387% and 870%, respectively). The upregulation of *SLC12A5* was not significantly different between GBM5-1F and GBM5-2F tumor cells after treatment (*p* > 0.05). In contrast, treatment with 2 mM NaVPA—3 mM NaDCA significantly reduced relative gene expression by 31% in GBM5-3F cells compared to its control. TMZ treatment did not affect *SLC12A5* expression in all three cells tested.

### 3.3. The Effect of NaVPA–NaDCA or TMZ on the CDH1 Expression in Female GBM Patients’ Tumor Primary Cells

*CDH1* expression was detected in all control cells, with the highest expression levels observed in GBM-2F and GBM-3F, showing no significant difference between them. These cells exhibited significantly higher expression compared to GBM-1F cells (*p* = 0.002).

**Figure 3:**
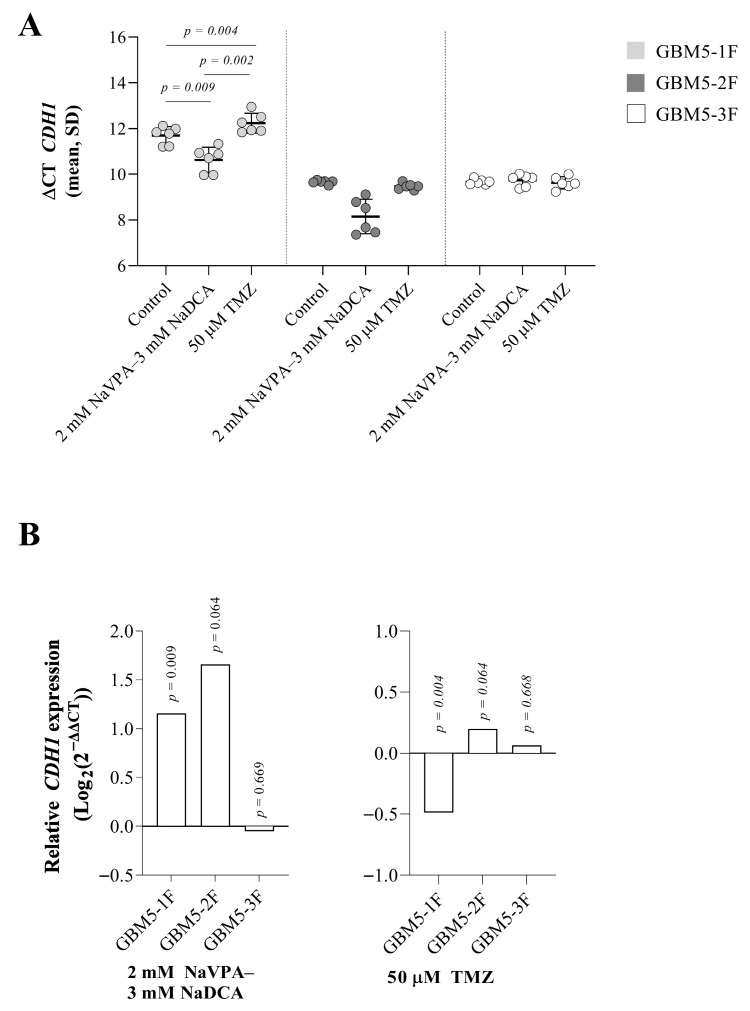
(**A**) The *CDH1* expression (ΔCT) of the GBM patient tumor control group, normalized to the *GAPDH*. (**B**) Relative *CDH1* expression (Log_2_(2^−ΔΔCT^)) of female GBM patient tumor cell treated groups. Exact *p*-values are given compared to the control.

The combination treatment significantly increased *CDH1* expression in GBM5-1F by 123%, while no significant changes in gene expression were detected in the other two patient cells. Upregulation of *CDH1* was significantly different (*p* = 0.0051) between GBM5-1F and GBM5-2F cells after treatment with NaVPA–NaDCA. TMZ only reduced CDH1 expression by 29% in GBM5-1F cells (Figure 3B).

### 3.4. The Effect of NaVPA–NaDCA or TMZ on the CDH2 Expression in Female GBM Patients’ Tumor Primary Cells

**Figure 4:**
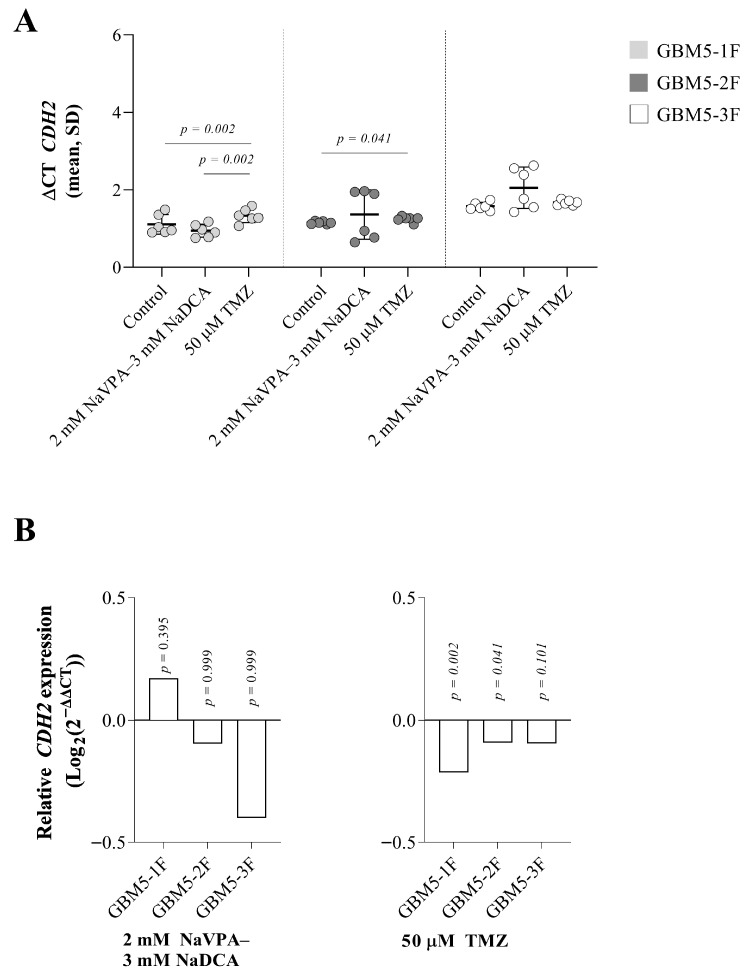
(**A**) *CDH2* expression (ΔCT) of GBM patient tumor control cell groups normalized to the *GAPDH* gene. (**B**) Relative *CDH2* expression (Log_2_(2^−ΔΔCT^)) of female GBM patient tumor groups tested. Exact p-values are given compared to the control.

The controls of GBM5-1F and GBM5-2F had similar *CDH2* expression, and their gene expression was significantly higher than that of GBM5-3F cells (*p* = 0.05). NaVPA–NaDCA treatment had no significant effect on *CDH2* expression in all three patients’ cells studied, but TMZ treatment significantly reduced relative gene expression in GBM5-1F and GBM5-2F cells (14% and 6%, respectively) and did not affect gene expression in GBM5-3F cells compared to the control (Figure 4B).

### 3.5. The Effect of NaVPA–NaDCA or TMZ on the EZH2 Expression in Female GBM Patients’ Tumor Primary Cells

There were differences in *EZH2* expression between the control cells tested. Comparing GBM-1F and GBM-2F cells, no difference in gene expression was found; however, GBM5-1F cells exhibit significantly lower gene expression than GBM5-3F cells (*p* = 0.002). GBM5-2F cells exhibit significantly lower *EZH2* expression compared to GBM5-3F cells.

**Figure 5:**
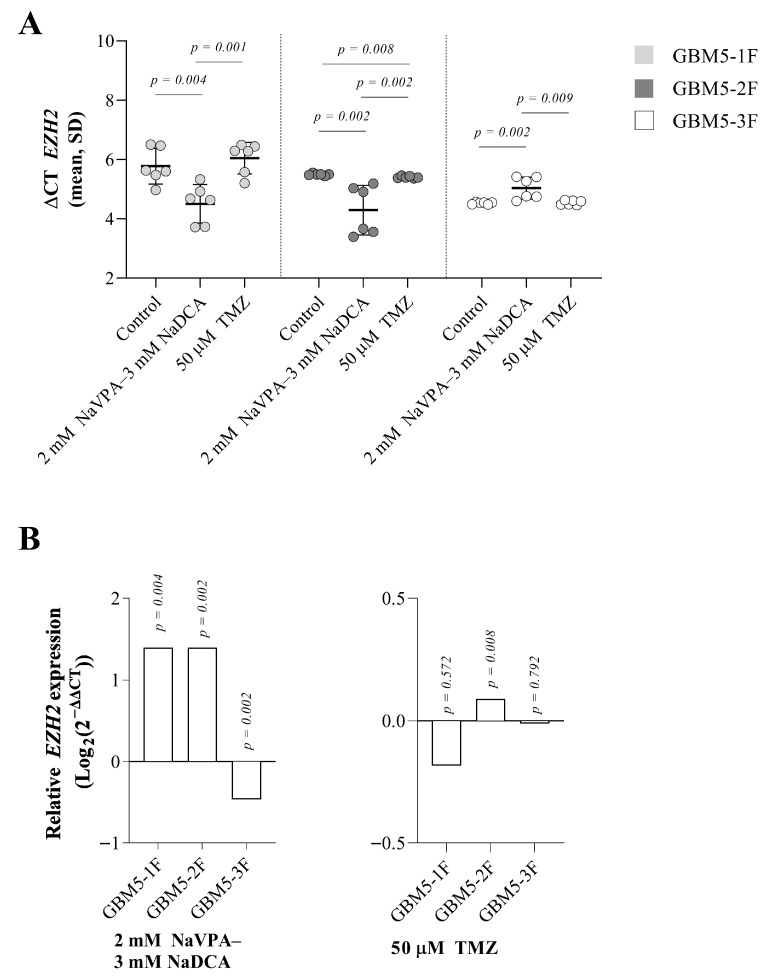
(**A**) The *EZH2* expression (ΔCT) of the GBM patient tumor control group was normalized to the *GAPDH*. (**B**) Relative *EZH2* expression (Log_2_(2^−ΔΔCT^)) in female GBM patient tumor cell treated groups. Exact *p*-values are given compared to the control.

Treatment with 2 mM NaVPA–3 mM DCA significantly increased relative *EZH2* expression in GBM5-1F cells by 168% and in GBM5-2F by 163%. (upregulation of *EZH2* was not significantly different between GBM5-1F and GBM5-2F cells after treatment with NaVPA–NaDCA), and significantly reduced GBM5-3F gene expression by 28% compared to the controls. TMZ significantly increased gene expression by 6% in GBM5-2F cells only (Figure 5B). After NaVPA–NaDCA treatment, *EZH2* expression was significantly higher in GBM5-1F and GBM5-2F and lower in GBM5-3F compared to the corresponding groups treated with TMZ.

### 3.6. The Effect of NaVPA–NaDCA or TMZ on the GFAP Expression in Female GBM Patients’ Tumor Primary Cells

The following differences in *GFAP* expression were observed between the control and test cells: gene expression was significantly lower in GBM5-2F compared to GBM5-1F (*p* = 0.002); the gene expression in GBM5-3F cells was significantly higher than in GBM5-2F (*p* = 0.002).

**Figure 6:**
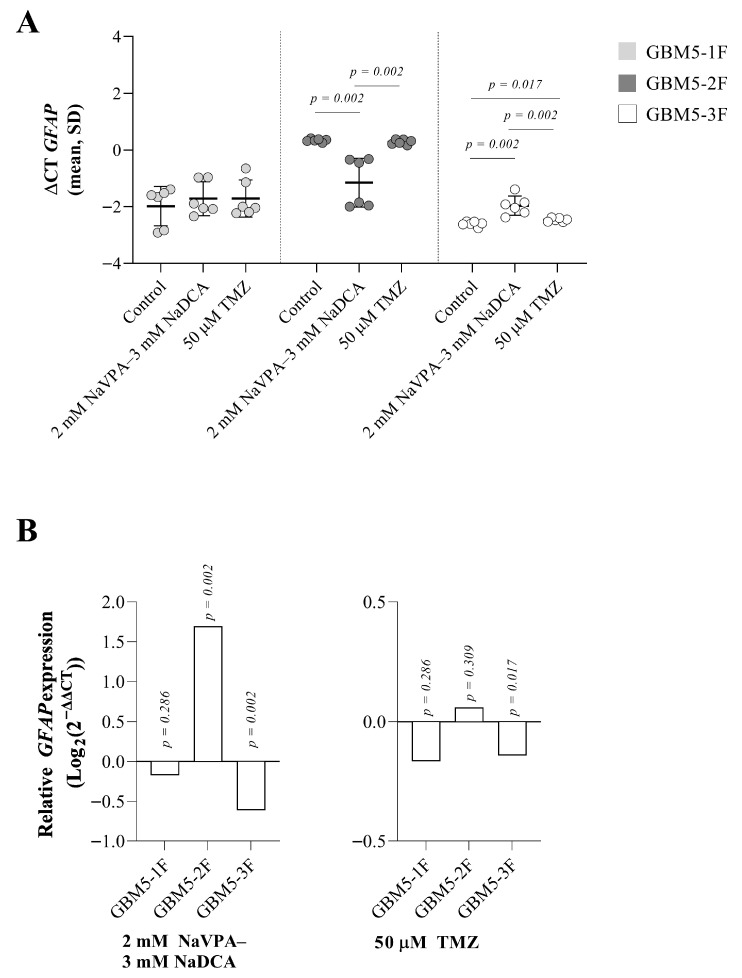
(**A**) The *GFAP* expression (ΔCT) of the GBM patient tumor control group was normalized to the *GAPDH* gene. (**B**) Relative *GFAP* expression (Log_2_(2^−ΔΔCT^)) of female GBM patient tumor treated cell groups. Exact *p*-values are given compared to the control.

Treatment with 2 mM NaVPA–3 mM NaDCA significantly upregulated *GFAP* expression by 224% in GBM5-2F and significantly downregulated gene expression by 35% in GBM5-3F cells compared with control levels and did not affect gene expression in GBM5-1F cells. TMZ treatment did not affect *GFAP* expression compared with control levels (Figure 6A,B).

## 4. Discussion

Glioblastoma (GBM) is a very aggressive type of glioma that practically recurs after first-line standard treatment with surgery, radiotherapy, and TMZ chemotherapy [62]. Five-year survivors of IDH-wild-type GBM patients represent a minority of the GBM population, and the differences in their molecular mechanisms compared to patients whose treatment was ineffective remain largely unexplored [63]. The invasive nature of GBM tumors is linked to the intercellular communication system used by cells to adapt to the microenvironment [64]. Characterizing the heterogeneity and invasiveness of patients’ tumors is challenging for research, and so is identifying targeted, personalized treatment approaches [7,65].

Experimental studies with GBM cell lines have shown that NaVPA–NaDCA exhibits anticancer effects on GBM by inhibiting tumor growth, invasion, proliferation, and angiogenesis, and its efficacy may be superior to that of TMZ. However, the efficacy of the investigational medicines depends on the GBM cell lines [43]. Macrophages, monocytes, and microglia cells are essential for GBM growth, and invasion is inhibited when these inflammatory and immune cells are suppressed [66]. However, the exact pathways involved in the tumor-sustaining process have not been characterized. Experimental and biomedical studies on T lymphocytes have shown that NaVPA–NaDCA inhibits inflammatory and immune pathways necessary for the growth of GBM; gene sequence analysis revealed a significant effect of NaVPA–NaDCA on inhibiting inflammatory mechanisms, leading to the downregulation of inflammation-related genes involved in the cytokine activity pathway, the inflammatory response pathway, and the IL-17 signaling pathway in mouse thymocytes [42]. The NaVPA–NaDCA has a significant anti-inflammatory effect on specific sets of genes involved in inflammation and immune response pathways, including cytokine activity, chemokine-mediated signaling, neutrophil chemotaxis, and lymphocyte chemotaxis, in T lymphocytes from women with SARS-CoV-2 infection and pneumonia [44].

In the study, we discuss below the differences in the expression of *SLC12A2*, *SLC12A5*, *CDH1*, *CDH2*, *EZH2*, and *GFAP* in primary GBM IDH-wild-type female cells (GBM5-1F, GBM5-2F, and GBM5-3F), and the differences in the response of the tested cells to the effects of the treatment with NaVPA–NaDCA compared to the impact of the treatment with TMZ.

The cation and chloride cotransporters, the Na-K-2Cl cotransporter (NKCC1) and the K-Cl cotransporter (KCC2), are essential for the determination of intracellular Cl^−^ concentrations in neurons and GBM cells [67]. High-grade glioblastoma cells accumulate intracellular chloride ([Cl^−^]i) ~10 times more than the average of grade II glioma and normal cortical cells [68]. *SLC12A2*, the gene encoding NKCC1, is expressed in GBMs in primary patient biopsies and patient-derived xenografts. *SLC12A5*, the gene that encodes KCC2, which carries Cl^−^ out of the cell, is poorly expressed in GBMs [51]. Treatment with NaVPA–NaDCA or TMZ significantly reduced *SLC12A2* expression in GBM5-3F cells, which had significantly higher gene expression than GBM5-2F cells in the control group. Treatment with the NaVPA–NaDCA increased *SLC12A2* expression in both GBM5-1F and GBM5-2F cells, whereas treatment with TMZ did not affect gene expression in the TMZ-treated respective cells. It was reported that 50 µM TMZ significantly increased *SLC12A2* expression in glioblastoma PBT24 and SF8628 cell lines [69]. Researchers hypothesized that NKCC1 activity in TMZ-treated GBM cells is stimulated via cell volume regulatory kinases and a WNK-mediated signaling pathway, which is vital in protecting gliomas from loss of cell volume and [Cl^−^]i in TMZ-treated cells [70,71], allowing GBM cells to rapidly adapt to the altered osmotic situation. Resistance to TMZ therapy is thought to be due to overexpression of the NKCC1 cotransporter, which enhances DNA repair mechanisms that protect against TMZ-induced apoptosis [72].

The expression of *SLC12A5* was significantly different in all the control cells tested. Specifically, cells exhibited GBM5-3F > GBM5-2F > GBM5-1F gene expression. NaVPA–NaDCA treatment significantly increased the relative expression of *SLC12A5* in GBM5-1F and GBM5-2F. In contrast, this treatment significantly reduced relative gene expression in GBM5-3F cells. TMZ treatment did not affect *SLC12A5* expression in all three cells tested. It was reported that TMZ treatment significantly increased *SLC12A5* expression in GBM PBT24 cells, while treatment of SF8628 cells did not affect gene expression [69]. However, the expression of *SLC12A2* should not only be considered unilaterally but also in terms of changes in the expression of *SLC12A5*. Evaluation of both cotransporter genes together after NaVPA–NaDCA treatment reveals that exposure significantly increases *SLC12A2* expression in GBM5-1F and GBM5-2F cells, thereby increasing Cl^−^ influx, concomitant with increased Cl^−^ efflux, as observed when *SLC12A5* is activated in GBM5-1F and GBM5-2F cells, respectively, suggesting an interplay between their functions. In GBM5-3F cells, NaVPA–NaDCA inhibited the expression of *SLC12A2* (Cl^−^ influx) while significantly reducing the expression of *SLC12A5* (Cl^−^ efflux). KCC2 is a vital cancer marker related to tumor cell apoptosis. The reduction in the intracellular K^+^ and Cl^−^ ion levels is related to the activation of caspases and triggers caspase cascade-related apoptosis mechanisms [73]. Furthermore, NKCC1 upregulation is associated with astrocyte swelling and a relatively high [Cl^−^]i content [74], as well as a GABAA receptor-mediated excitatory response that facilitates seizure onset [75]. KCC2, conversely, is a neuron-specific Cl^−^ extruder that uses a K^+^ gradient to maintain low [Cl^−^]i levels and ensure the proper functioning of postsynaptic GABAA receptors. Low KCC2 expression and function are hallmarks of epileptic brain disorders. The effect of drugs that activate KCC2 function in GBM is important as a potential new therapeutic target for GBM [76].

The majority of GBM did not express *CDH1* [77]. It is a rare occurrence to encounter malignant gliomas with E-cadherin expression [78]. GBMs without epithelial phenotypes exhibit a substantial alteration of the cytoskeleton, which differs from the classical EMT of epithelial tumors, characterized by a shift in E-cadherin away from N-cadherin [79,80]. In our study, *CDH1* expression was detected in all control cells, with the highest expression levels observed in GBM-2F and GBM-3F. These cells exhibited significantly higher *CDH1* expression compared to GBM-1F. The VPA-NaDCA significantly increased *CDH1* expression in GBM5-1F only. TMZ only reduced *CDH1* expression in GBM5-1F cells. GBM5-1F and GBM5-2F exhibited similar *CDH2* expression, and their gene expression was higher than that of GBM5-3F cells. VPA–NaDCA treatment did not affect *CDH2* expression in the cells studied; however, TMZ treatment significantly reduced relative gene expression in GBM5-1F and GBM5-2F cells, but did not affect gene expression in GBM5-3F cells. The EMT process in gliomas may be exacerbated by enhanced *CDH2* expression, which is associated with unfavorable prognostic outcomes. *CDH2* was expressed in the majority of glioma cases, with no expression found in 10.3% of high-grade GBM [81].

The following research areas are the GBM markers *EZH2* and *GFAP* gene expression. The histone methyltransferase *EZH2* is often overexpressed in GBM, regulating gene transcription and promoting tumor genesis by inhibiting the function of tumor suppressor genes [82,83]. EZH2 facilitates glioma proliferation, migration, and invasion [58]. The differences in *EZH2* expression between the control cells are significant in the tested cells: GBM53F > GBM2F or GBM5-1F. Treatment with NaVPA–NaDCA significantly increased relative *EZH2* expression in GBM5-1F and GBM5-2F and reduced GBM5-3F cell gene expression considerably. TMZ significantly increased gene relative expression in GBM5-2F cells only. The EZH2-SLC12A5 axis in GBM lays a new foundation for the clinical translation of NaVPA–NaDCA treatment, offering new insights for precision GBM therapy. Our study demonstrates that NaVPA–NaDCA can upregulate *SLC12A5* expression, suggesting that this beneficial effect may counteract the effects of *EZH2* in GBM cells.

The following significant differences in *GFAP* expression were observed between the control: GBM5-2F < GBM5-1F and GBM5-3F. Treatment with NaVPA–NaDCA significantly upregulated *GFAP* expression in GBM5-2F, significantly downregulated it in GBM5-3F, and did not affect gene expression in GBM5-1F cells. TMZ treatment did not affect *GFAP* expression in the tested cells. The *Ezh2^cKO^* mouse model demonstrates that the depletion of Ezh2 from astrocytes significantly increases GFAP expression. Furthermore, experimental studies indicate that the loss of EZH2 in astrocytes leads to disruption of the blood–brain barrier (BBB) [84].

A limitation of the study could be that the number of primary GBM cells studied was small and limited to women. Therefore, studies are needed on the impact of gender differences on treatment effects. Another limitation of the study was that it did not account for other mechanisms that influence protein levels or activity, thereby limiting the assessment of the mechanism to mRNA. However, the activity of the proteins studied is influenced by many mechanisms other than gene expression (mRNA). Regarding the lack of studies on protein levels, it is worth noting that the correlation between differentially expressed mRNA and mRNA/protein levels of the same gene is a topic of debate. Genome-wide correlation between mRNA and protein expression levels is notoriously poor; typically, 0–50% of mRNA levels correspond to protein expression, with a correlation between mRNA and protein expression levels of around 40% explanatory power across many studies. Correlations between differentially expressed mRNA profiles were low and even negative [85,86,87,88].

The studies clearly show that GBM tissue cells exhibit a polymorphic response to tested treatment, indicating the importance of assessing the effects of chemotherapeutic agents before treatment is administered to personalize treatment. NaVPA and NaDCA have been used for treatment for decades, have the potential to cross the BBB, and their safety profile and blood levels are well known, encouraging further testing for new GBM therapeutic indications. Our in vitro studies demonstrate that NaVPA–NaDCA treatment is superior to TMZ treatment in certain respects.

## 5. Conclusions

1. The combination of sodium valproate and sodium dichloroacetate, as well as temozolomide, had individual impacts in vitro on the *SLC12A2*, *SLC12A5*, *CDH1*, *CDH2*, *EZH*, and *GFAP* expressions in GBM5-1, GBM5-2F, and GBM5-3F primary cells of GBM wild-type tumors.
2. When assessing the changes in *SLC12A2* and *SLC12A5*, *CDH1* and *CDH2*, or *EZH2* and *GFAP* expressions after treatment with NaVPA–NaDCA or TMZ, it was found that NaVPA–NaDCA could have an advantage of antitumor activity, and that may be more effective than TMZ, but the effect is individual.
3. To determine the differences in the efficacy of NaVPA–NaDCA and TMZ, further preclinical studies are needed to investigate their effects on the expression and function of GBM pathogenesis-relevant proteins, as well as their effects on GBM carcinogenesis pathways in the research of individualized treatment.

## 6. Patents

The combination of VPA and NaDCA products is for the treatment of cancer (Official bulletin of the state patent bureau of the Republic of Lithuania, No. 6874, filling date 17 April 2020 [89]), A European patent application has been submitted (European patent application No. 21168796.7, filing date 16 April 2021 [90]).

## Data Availability

The data presented in this study are available on request from the corresponding author.

## Supplementary Materials

The following supporting information can be downloaded at: https://www.mdpi.com/article/10.3390/pharmaceutics17091161/s1, Table S1: SLC12A2 and GAPDH expression data from female GBM patient cells in control and treated groups; Table S2: SLC12A5 and GAPDH expression data from female GBM patient cells in the control and treated groups; Table S3: *CDH1* and *GAPDH* expression data from female GBM patient tumor cells in the control and treated groups; Table S4: CDH2 and GAPDH expression data from female GBM patient tumor cells in the control and treated groups; Table S5: *EZH2* and *GAPDH* expression data from female GBM patient tumor cells in the control and treated groups; Table S6: *GFAP* and *GAPDH* expression data from female GBM patient tumor cells in the control and treated groups.

## Abbreviations

The following abbreviations are used in this manuscript:

BBB	blood–brain barrier
CDH1	E-cadherin
CDH2	N-cadherin
DCA	dichloroacetate
EZH2	enhancer of zest homolog 2
GABAA	γ-aminobutyric acid A receptor
GBM	glioblastoma
GFAP	glial fibrillary acidic protein
GPI	glycophosphatidylinositol
HDAC	histone deacetylase
IDH	isocitrate dehydrogenase
KCC2	K-Cl cotransporter (SLC12A5)
MGMT	methylated-DNA-protein-cysteine methyltransferase
NaDCA	sodium dichloroacetate
NaVPA	sodium valproate
NaVPA–NaDCA	sodium valproate and sodium dichloroacetate combination
NKCC1	Na-K-2Cl cotransporter (SLC12A2)
OS	overall survival
PDH	pyruvate dehydrogenase
PDK	pyruvate dehydrogenase kinase
PGK1	phosphoglycerate kinase 1
SLC5A8	sodium-coupled monocarboxylate transporter
TMZ	temozolomide
WNK1-OSR1-NKCC1	lysine-deficient protein kinase-1—oxidative stress responsive 1—NKCC1 pathway
