# Supplementary material for: Comparison of the Effect of the Combination of Sodium Valproate and Sodium Dichloroacetate on the Expression of SLC12A2, SLC12A5, CDH1, CDH2, EZH2, and GFAP in Primary Female Glioblastoma Cells with That of Temozolomide
Source: Pharmaceutics. 2025 Sep 4;17(9):1161. doi: 10.3390/pharmaceutics17091161 (PMC12473538; doi:10.3390/pharmaceutics17091161)
Supplement: Supplementary file 1 [file pharmaceutics-17-01161-s001.zip › pharmaceutics-3772928-supplementary.pdf]

Supplementary Materials: Tables of Supplementary Data (Tables S1–S6)

Table S1. *SLC12A2* and *GAPDH* expression data from female GBM patient cells in control and treated groups

Study Groups	n	Indicator. Mean ± SD			ΔΔCT	$2^{-\Delta\Delta CT}$
		CT		ΔCT		
		SLC12A2	*GAPDH*			
**GBM5-1F**						
Control	6	23.47 ± 0.86	18.02 ± 0.63	5.44 ± 0.91		
2 mM NaVPA–3 mM NaDCA-treated	6	23.34 ± 1.01	18.76 ± 0.65	4.57 ± 1.08	-0.87	2.27
50 µM TMZ	6	23.32 ±0.90	17.97 ± 0.53	5.35 ± 0.95	-0.10	1.29
**GBM5-2F** [c]						
Control	6	24.55 ± 0.11	19.17 ± 0.31	6.39 ± 0.11[b]		
2 mM NaVPA–3 mM NaDCA-treated	6	24.92 ± 0.98	19.85 ± 1.15	5.07 ± 0.98 [a]	-1.32	2.99
50 µM TMZ	6	24.69 ±0.14	18.40 ± 0.41	6.29 ± 0.14	-0.10	1.07
**GBM5-3F** [c]						
Control	6	25.11±0.08	19.09±0.16	6.03±0.08		
2 mM NaVPA–3 mM NaDCA-treated	6	25.50±0.55	18.55±0.48	6.95±0.55 [a]	0.93	0.56
50 µM TMZ	6	25.48±0.10	19.09±0.26	6.39±0.10 [a]	0.37	0.78

[a] $p < 0.01$ compared to control; [b] $p < 0.01$ compared to GBM5-3F control - Mann-Whitney U test.
[c] $p < 0.001$ – Kruskal-Wallis test.

Table S2. *SLC12A5* and *GAPDH* expression data from female GBM patient cells in the control and treated groups

Study Groups	n	Indicator. Mean ± SD			ΔΔCT	$2^{-\Delta\Delta CT}$
		CT		ΔCT		
		SLC12A5	*GAPDH*			
**GBM5-1F** [d]						
Control	6	30.76 ± 0.67	18.02 ± 0.63	12.74 ± 0.67 [b,c]		
2 mM NaVPA–3 mM NaDCA-treated	6	29.73 ± 0.72	18.76 ± 0.65	10.98 ± 0.73 [a]	-1.76	3.87
50 µM TMZ	6	31.67 ±0.29	17.97 ± 0.53	13.70 ±0.29	0.96	0.52
**GBM5-2F** [d]						
Control	6	33.27 ± 0.19	19.17 ± 0.31	15.10 ± 0.19 [b]		
2 mM NaVPA–3 mM NaDCA-treated	6	31.98 ± 1.09	19.85 ± 1.15	12.14 ± 1.09 [a]	-2.97	9.77
50 µM TMZ	6	33.12 ±0.40	18.40 ± 0.41	14.72 ±0.40 [d]	-0.38	1.34
**GBM5-3F** [e]						
Control	6	31.03 ± 0.16	19.09 ± 0.16	11.94 ± 0.17		
2 mM NaVPA–3 mM NaDCA-treated	6	31.04 ± 0.28	18.55 ± 0.48	12.49 ± 0.29 [a]	0.55	0.69
50 µM TMZ	6	31.00 ± 0.28	19.09 ± 0.26	11.91 ± 0.28	-0.03	1.03

[a] $p < 0.01$ compared to control; [b] $p < 0.01$ compared to GBM5-3F control; [c] $p < 0.01$ compared to GBM5-2F - Mann-Whitney U test.
[d] $p < 0.001$; [e] $p < 0.01$ – Kruskal-Wallis test.

Table S3. *CDH1* and *GAPDH* expression data from female GBM patient tumor cells in the control and treated groups

Study Groups	n	Indicator. Mean ± SD			ΔΔCT	$2^{-ΔΔCT}$
		CT		ΔCT		
		CDH1	*GAPDH*			
**GBM5-1F** [d]						
Control	6	30.09 ± 0.39	18.39 ± 0.26	11.70 ± 0.39 [b,c]		
2 mM NaVPA–3 mM NaDCA-treated	6	30.05 ± 0.56	19.42 ± 0.13	10.63 ± 0.56 [a]	-1.07	2.23
50 µM TMZ	6	30.47 ±0.43	18.24 ± 0.24	12.23 ±0.43 [a]	0.54	0.71
**GBM5-2F** [d]						
Control	6	27.82 ± 0.08	18.16 ± 0.28	9.65 ± 0.08		
2 mM NaVPA–3 mM NaDCA-treated	6	28.00 ± 0.75	19.85 ± 1.15	8.15 ± 0.75	-1.50	3.15
50 µM TMZ	6	27.86 ±0.14	18.40 ± 0.41	9.46 ± 0.14	-0.19	1.15
**GBM5-3F**						
Control	6	28.75 ± 0.12	19.09 ± 0.16	9.66 ± 0.12		
2 mM NaVPA–3 mM NaDCA-treated	6	28.28 ± 0.26	18.55 ± 0.48	9.74 ± 0.26	0.08	0.96
50 µM TMZ	6	28.71 ± 0.27	19.09 ± 0.26	9.62 ± 0.27	-0.04	1.04

[a] $p < 0.01$ compared to control; [b] $p < 0.01$ compared to GBM5-3F control; [c] $p < 0.01$ compared to GBM5-2F - Mann-Whitney U test.
[d] $p < 0.001$ – Kruskal-Wallis test.

Table S4. *CDH2* and *GAPDH* expression data from female GBM patient tumor cells in the control and treated groups

Study Groups	n	Indicator. Mean ± SD			ΔΔCT	$2^{-ΔΔCT}$
		CT		ΔCT		
		CDH2	*GAPDH*			
**GBM5-1F** [d]						
Control	6	19.51 ± 0.25	18.39 ± 0.26	1.11 ± 0.25 [c]		
2 mM NaVPA–3 mM NaDCA-treated	6	20.36 ± 0.17	19.42 ± 0.13	0.95 ± 0.17	-0.16	1.13
50 µM TMZ	6	19.57 ±0.18	18.24 ± 0.24	1.33 ± 0.18 [a]	0.22	0.86
**GBM-2F**						
Control	6	19.31 ± 0.04	18.16 ± 0.28	1.15 ± 0.04 [c]		
2 mM NaVPA–3 mM NaDCA-treated	6	21.21 ± 0.64	19.85 ± 1.15	1.36 ± 0.64	0.21	0.94
50 µM TMZ	6	19.65 ±0.08	18.40 ± 0.41	1.25 ± 0.08 [b]	0.09	0.94
**GBM5-3F**						
Control	6	20.66 ± 0.11	19.09 ± 0.16	1.58 ± 0.11		
2 mM NaVPA–3 mM NaDCA-treated	6	20.61 ± 0.53	18.55 ± 0.48	2.06 ± 0.53	0.48	0.76
50 µM TMZ	6	20.76 ± 0.07	19.09 ± 0.26	1.67 ± 0.07	0.09	0.94

[a] $p < 0.01$ compared to control; [b] $p < 0.05$ compared to control; [c] $p < 0.01$ compared to GBM5-3F control - Mann-Whitney U test.
[d] $p < 0.05$ – Kruskal-Wallis test.

Table S5. *EZH2* and *GAPDH* expression data from female GBM patient tumor cells in the control and treated groups

Study Groups	n	Indicator. Mean ± SD			ΔΔCT	$2^{-\Delta\Delta CT}$
		CT		ΔCT		
		EZH2	*GAPDH*			
**GBM5-1F** [c]						
Control	6	23.80 ±0.62	18.02 ± 0.63	5.77 ± 0.62 [b]		
2 mM NaVPA–3 mM NaDCA-treated	6	23.27 ±0.75	18.76 ± 0.65	4.51 ± 0.65 [a]	-1.26	2.68
50 µM TMZ		23.86 ±0.54	17.97 ± 0.53	6.05 ± 0.53	0.27	0.88
**GBM5-2F** [d]						
Control	6	23.65 ±0.04	19.17 ± 0.31	5.49 ± 0.04 [b]		
2 mM NaVPA–3 mM NaDCA-treated	6	24.14 ±0.83	19.85 ± 1.15	4.29 ± 0.83 [a]	-1.20	2.63
50 µM TMZ	6	23.80 ±0.04	18.40 ± 0.41	5.40 ± 0.04 [a]	-0.09	1.06
**GBM5-3F** [d]						
Control	6	23.62 ± 0.03	19.09 ± 0.16	4.53 ± 0.03		
2 mM NaVPA–3 mM NaDCA-treated	6	23.58 ± 0.37	18.55 ± 0.48	5.04 ± 0.37 [a]	0.51	0.72
50 µM TMZ	6	23.63 ± 0.08	19.09 ± 0.26	4.54 ± 0.08	0.01	0.99

[a] $p < 0.01$ compared to control; [b] $p < 0.01$ compared to GBM5-3F control - Mann-Whitney U test.
[c] $p < 0.01$; [d] $p < 0.001$ – Kruskal-Wallis test.

Table S6. *GFAP* and *GAPDH* expression data from female GBM patient tumor cells in the control and treated groups

Study Groups	n	Indicator. Mean ± SD			ΔΔCT	$2^{-\Delta\Delta CT}$
		CT		ΔCT		
		GFAP	*GAPDH*			
**GBM5-1F**						
Control	6	16.04 ±0.82	18.02 ± 0.63	-1.98 ± 0.69 [c]		
2 mM NaVPA–3 mM NaDCA-treated	6	17.05 ±0.68	18.76 ± 0.65	-1.72 ± 0.60	0.28	0.90
50 µM TMZ	6	16.26 ±0.71	17.97 ± 0.53	-1.71 ± 1.42	0.27	0.89
**GBM5-2F** [d]						
Control	6	18.50 ±0.06	19.17 ± 0.31	0.34 ± 0.06		
2 mM NaVPA–3 mM NaDCA-treated	6	18.69 ±0.86	19.85 ± 1.15	-1.15 ± 0.86 [a]	-1.49	3.24
50 µM TMZ	6	18.68 ±0.09	18.40 ± 0.41	0.28 ± 0.09	-0.06	1.04
GBM5-3F [e]						
Control	6	16.48 ± 0.08	19.09 ± 0.16	-2.60 ± 0.08 [c]		
2 mM NaVPA–3 mM NaDCA-treated	6	16.59 ± 0.34	18.55 ± 0.48	-1.96 ± 0.34 [a]	0.64	0.65
50 µM TMZ	6	16.63 ± 0.07	19.09 ± 0.26	-2.46 ± 0.07 [b]	0.14	0.91

[a] $p < 0.01$ compared to control; [b] $p < 0.01$ compared to control; [c] $p < 0.01$ compared to GBM5-2F control - Mann-Whitney U test.
[d] $p < 0.0001$; [e] $p < 0.001$ – Kruskal-Wallis test.
